# Metabolomic Alterations Do Not Induce Metabolic Burden in the Industrial Yeast M2n[pBKD2-*Pccbgl1*]-C1 Engineered by Multiple δ-Integration of a Fungal β-Glucosidase Gene

**DOI:** 10.3389/fbioe.2019.00376

**Published:** 2019-11-28

**Authors:** Lorenzo Favaro, Lorenzo Cagnin, Laura Corte, Luca Roscini, Fabio De Pascale, Laura Treu, Stefano Campanaro, Marina Basaglia, Willem H. van Zyl, Sergio Casella, Gianluigi Cardinali

**Affiliations:** ^1^Department of Agronomy Food Natural Resources Animals and Environment (DAFNAE), University of Padova, Legnaro, Italy; ^2^Department of Pharmaceutical Sciences-Microbiology, University of Perugia, Perugia, Italy; ^3^Department of Biology, University of Padova, Padova, Italy; ^4^Department of Microbiology, Stellenbosch University, Matieland, South Africa; ^5^Department of Chemistry, Biology and Biotechnology, Centre of Excellence on Nanostructured Innovative Materials (CEMIN), University of Perugia, Perugia, Italy

**Keywords:** lignocellulosic ethanol, metabolic burden, metabolomic fingerprint, Fourier transform infrared spectroscopy, stress response

## Abstract

In the lignocellulosic yeast development, metabolic burden relates to redirection of resources from regular cellular activities toward the needs created by recombinant protein production. As a result, growth parameters may be greatly affected. Noteworthy, *Saccharomyces cerevisiae* M2n[pBKD2-*Pccbgl1*]-C1, previously developed by multiple δ-integration of the β-glucosidase *BGL3*, did not show any detectable metabolic burden. This work aims to test the hypothesis that the metabolic burden and the metabolomic perturbation induced by the δ-integration of a yeast strain, could differ significantly. The engineered strain was evaluated in terms of metabolic performances and metabolomic alterations in different conditions typical of the bioethanol industry. Results indicate that the multiple δ-integration did not affect the ability of the engineered strain to grow on different carbon sources and to tolerate increasing concentrations of ethanol and inhibitory compounds. Conversely, metabolomic profiles were significantly altered both under growing and stressing conditions, indicating a large extent of metabolic reshuffling involved in the maintenance of the metabolic homeostasis. Considering that four copies of *BGL3* gene have been integrated without affecting any parental genes or promoter sequences, deeper studies are needed to unveil the mechanisms implied in these metabolomic changes, thus supporting the optimization of protein production in engineered strains.

## Introduction

Today, bioethanol as major biofuel is mostly obtained from corn, wheat, and sugarcane (Mohanty and Swain, 2019). However, the ideal substrate for bioethanol production is non-edible lignocellulosic biomass, like energy crops, spruce or birch, or agricultural by-products (Zhang, 2019). Lignocellulose represents a complex recalcitrant matrix that requires costly pre-treatment and enzyme supplementation to yield fermentable sugars from embedded polysaccharides. During pre-treatment, lignocellulosic material is partly degraded to inhibitory compounds, such as furans, weak acids and phenolics, which are toxic to the microbial metabolism. These inhibitors can slow down or even stop the fermentation, limiting the process efficiency (Almeida et al., 2007).

*Saccharomyces cerevisiae* is unable to directly ferment the cellulose fibers exposed after lignocellulose pre-treatment. Therefore, to convert cellulose into glucose, industrial bioethanol production requires the use of expensive commercial cellulases, which negatively impacts the feasibility of the overall process. Consequently, the development of engineered *S. cerevisiae* strains able to produce one or more cellulolytic enzymes is required. Such new phenotypic traits can be obtained by engineering robust yeast strains to produce one or more heterologous cellulases (Van Zyl et al., 2007). However, engineering industrial strains for sufficient production of functional cellulases still remains a major challenge (Van Zyl et al., 2007; Den Haan et al., 2015). Although noteworthy advancement has been made, a deeper understanding of the mechanisms governing heterologous protein production in yeast will be crucial for developing more efficient protein production systems.

The expression of cellulase genes can induce a stressful condition, known as metabolic burden, that may impair the metabolic performances of the recombinant strain (Van Rensburg et al., 2012; Ding et al., 2018; Wei et al., 2018). The concept of metabolic burden arose from previous investigations in this area and became a keystone in yeast synthetic biology and metabolic engineering (Wu et al., 2016; Zahrl et al., 2019). In the case of lignocellulosic yeast strains development, metabolic burden often relates to additional energetic costs caused by the synthesis of recombinant proteins or to the effects of competition for limited transcriptional and translational resources required in protein production and secretion. As a result, growth parameters, such as biomass yield, growth and specific substrate consumption rate, may be greatly affected. Furthermore, part of the available sugars may be redirected from desired ethanol to unwanted glycerol and acetate due to redox imbalances (Van Rensburg et al., 2012; Ding et al., 2018).

Both the strategy selected for the genetic engineering (episomal plasmid vs. chromosomal integration) and host strain (laboratory vs. industrial or natural yeast) are considered among the major players of metabolic burden related to heterologous proteins production. Nevertheless, the occurrence and the extent of metabolic burden is not yet well-understood (Favaro et al., 2012, 2015; Karim et al., 2013; Wu et al., 2016; Liu et al., 2017; Papapetridis et al., 2018; Li et al., 2019; Zahrl et al., 2019) and further studies are imperative to develop mitigation strategies and increase the recombinant strain performances in the biotechnological industry.

In a recent paper we demonstrated that the industrial yeast M2n[pBKD2-*Pccbgl1*]-C1 with multiple δ-integration of a specific β-glucosidase gene did not show any detectable metabolic burden in terms of ethanol production and yield of the recombinant strain vs. that of the parental yeast (Cagnin et al., 2019). This result poses the problem on whether the metabolic burden simply does not exist or cannot be detected by measuring only parameters such as the ethanol production, growth rate, and biomass yield. In the latter case, a more detailed comparison of the physiological status of the recombinant and of the parental host should shed light on this controversial situation. It has been already demonstrated that the viability and the metabolomic fingerprint of yeast cells subject to stress conditions are not necessarily related and vary in a strain-specific manner (Favaro et al., 2016; Colabella et al., 2017). This indicates that the metabolome could be perturbed in a significant way, without affecting physiological parameters. These evidences imply that metabolic burden could be present but not detectable in the case of M2n[pBKD2-*Pccbgl1*]-C1 and suggest metabolomic fingerprinting to elucidate this phenomenon.

Fourier-transform infrared (FTIR) spectroscopy was introduced in the early nineties to provide the molecular fingerprint of microorganisms describing the metabolic state of whole cells in a specific experimental condition (Helm et al., 1991; Naumann et al., 1991; Corte et al., 2015a). FTIR spectroscopy is an high-throughput technique to achieve massive and rapid information at very low running costs (Timmins et al., 1998; Kohler et al., 2015). A powerful application of this technique is the characterization of the physiological status of microbial cells under stress, indicating the type of molecules involved in a differential response and quantifying the extent of such stress response(s) (Aguilera et al., 2006; Dean et al., 2010; Mihoubi et al., 2017; Nguyen et al., 2017; Canal et al., 2019). Moreover, a FTIR-based assay has been developed in our laboratory for the rapid evaluation of the stress-induced cell status in response to different conditions, with the rationale that stress conditions can alter the cell metabolome long before cellular death occurs (Corte et al., 2010, 2015b; Favaro et al., 2016; Moktaduzzaman et al., 2016).

This work aims to test the hypothesis that the metabolic burden and the metabolomic perturbation induced by the δ-integration of a yeast strain, could differ significantly. For this purpose, the location and the extent of the δ -integration were characterized and a series of comparisons between the parental and the recombinant strains were performed to assess their metabolic performances and metabolomic alterations in different conditions typical of the bioethanol industry. Moreover, a novel procedure has been introduced to obtain robust statistical significance of the observed metabolomic variations.

## Materials and Methods

### Cultures and Growth Conditions

The yeast strains employed in this work are: *S. cerevisiae* M2n, an industrial distillery yeast (Favaro et al., 2015) and M2n[pBDK1-*BGL3*]-C1, recently developed through the delta-integration of the *BGL3* gene of *Phanaerochaete chrysosporium* into the M2n chromosomes (Cagnin et al., 2019).

For the incubation under aerobic condition, each pre-culture was inoculated at an optical density at 600 nm (OD_600_) = 0.2 in 500 mL bottles containing 50 mL of fresh YNB (Yeast Nitrogen Base, Sigma-Aldrich, Saint Louis, MO, USA) supplemented with either 0.1% glucose and 1.8% glycerol or the equivalent amount of cellobiose (2.05%) and grown at 25°C under shaking at 150 rpm. Growth under oxygen-limited condition was carried out by inoculating with pre-cultures at OD_600_ = 0.2 in 100 mL bottles containing 10 mL of fresh YNB supplemented with either 2.0% glucose or the equivalent amount of cellobiose (2.05%). Bottles were sealed with rubber stoppers, incubated at 25°C and mixed at 150 rpm on a magnetic stirrer. Syringe needles pierced through the bottle stopper served for sampling purposes and carbon dioxide release.

Cell growth was monitored by determining OD_600_. At targeted growth phases (lag, early and late exponential, early and late stationary, and death stages), cells suspensions were sampled and prepared for FTIR analysis as detailed in “Metabolomic fingerprint of growth” paragraph.

Cells suspensions for the FTIR based bioassay were prepared inoculating each strain at OD_600_ = 0.2 in YPD medium (yeast extract 1%, peptone 1%, and dextrose 2% Difco Laboratories, USA) and grown by shaking at 200 rpm for 18 h at 25°C. Each suspension was sampled and prepared for FTIR based bioassay, as detailed below in the “Cell stressing” paragraph.

### Genomic DNA Extraction and Library Sequencing

Genomic DNA was extracted from overnight yeast cultures by zymolyase digestion and standard phenol-chloroform extraction (Treu et al., 2014). A combined sequencing approach was then applied using Illumina and Oxford Nanopore MinION single molecule sequencers. Illumina library was generated using the TruSeq DNA PCR-Free Library Prep Kit (Illumina Inc., San Diego CA) and Covaris S2 (Woburn, MA) for a 550-bp average fragment size. Library was loaded onto the flow cell provided in the NextSeq 500 Reagent kit v2 (150 cycles) (Illumina Inc., San Diego CA) and sequenced on a NextSeq 500 (Illumina Inc., San Diego CA) platform with a paired-end protocol and read lengths of 151 bp at the CRIBI Biotechnology Center (Padova, Italy). Nanopore library was prepared according to SQK-LSK109 ligation sequencing kit and sequenced on a FLO-MIN106 R9 flowcell.

### Next Generation Sequencing Data Analysis

The genome assemblies of M2n and C1 strains were performed with a *de novo* approach by in house developed pipeline for combined Nanopore-Illumina sequences analysis. Briefly, the long reads were corrected with the Canu software (Koren et al., 2017) and assembled with SMART*denovo* (Ruan, 2019). The obtained contigs were polished with Pilon software (Walker et al., 2014) using the independent high-quality Illumina sequences and ordered according to the *S. cerevisiae* S288c reference genome using Mauve software (Darling et al., 2010). A whole genome alignment was then obtained with nucmer (Kurtz et al., 2004) to highlight genome completeness. The final genome of *S. cerevisiae* C1 was used to create a local database for BLAST analysis. The integrated genes *BGL3, KanMX (kanamycin resistance)* and *PGK1* promoter and terminator sequences were used as queries for BLAST search to determine the copy number of integrated cassettes. Furthermore, plasmid backbone of the integrative plasmid used to engineer C1 for the expression of *BGL3* was found in three copies in all the C1 genome: two copies between the first and second *BGL3* integrated cassette and a copy between the second and third *BGL3* integrated cassette. Raw reads of *S. cerevisiae* M2n and C1 was deposited at GenBank under the BioProject accession number PRJNA573579.

### Stress Inducing Agents

Formic acid, acetic acid, furfural, 5-hydroxymethyl-2-furaldehyde (HMF), cinnamic acid, and coniferyl aldehyde have been selected as representative of three important groups of inhibitors (aliphatic acids, furaldehydes, and aromatic compounds) of lignocellulose hydrolysates. Inhibitors were all obtained from Sigma (Sant Louis, MO, USA) and formulated into four inhibitor mixtures at increasing concentrations in distilled sterile water, as detailed in Table 1. Each inhibitor concentration has been chosen based on literature data (Martin and Jönsson, 2003; Favaro et al., 2016). Ethanol has been tested at increasing concentration of 7.5, 15, 25, and 30% (v/v). Each inhibitors mixture was also tested in absence of ethanol and at ethanol concentration of 7.5%.

**Table 1:** Inhibitor mixtures used in this study.

**Inhibitors**	**Concentration (mM)**			
	**A**	**B**	**C**	**D**
Acetic acid	20.00	40.00	60.00	120.00
Formic acid	7.00	13.00	20.00	27.00
Cinnamic acid	0.25	0.51	0.76	1.00
Coniferyl aldehyde	0.25	0.50	0.80	1.00
Furfural	7.00	14.00	22.00	29.00
HMF	6.50	13.00	19.00	25.00

### FTIR Analysis

#### Metabolomic Fingerprint of Growth

Cells suspensions, prepared as detailed in “Cultures and growth conditions” section, were centrifuged (5 min at 5,300 × g), washed twice with distilled sterile water and re-suspended in 1.5 mL HPLC (High Performance Liquid Chromatography) grade water to the final concentration of 2.5 × 10^8^ cells mL^−1^. For each culture, 105 μL volume was sampled for three independent FTIR readings (35 μL ml each, according to the technique suggested by Essendoubi et al. (2005).

#### Cell Stress

A FTIR based assay for stress response analysis was carried out according to the procedure proposed by Corte et al. (2010). Briefly, each cells suspension was centrifuged, washed twice with distilled sterile water and re-suspended in HPLC grade water to obtain an optical density of OD_600_ = 50. Each cell suspension was distributed in 1.7 mL polypropylene tubes, one for each tested concentration of the chemicals. In each tube were pipetted 500 μL cell suspension and 500 μL double concentrate solution of the stress inducing agent, in order to obtain the final concentrations of the chemicals reported in Table 1 and a uniform cell density at OD_600_ = 25. Controls (0% ethanol concentration, no inhibitor mixtures) was obtained by re-suspending cells directly in distilled sterile water. All tests were carried out in triplicate. The polypropylene tubes were incubated 1h at 25°C in a shaking incubator set at 50 rpm. After the incubation, cells were centrifuged (5 min at 5,300 × g), washed twice with distilled sterile water and resuspended in 1.5 mL HPLC grade water to the final concentration of 2.5 × 10^8^ cells mL^−1^. For each culture, 105 μL volume was sampled for three independent FTIR readings (35 μL each, according to the technique suggested by Essendoubi et al. (2005).

#### Spectra Pre-processing

FTIR measurements were performed in transmission mode. All spectra were recorded in the range between 4,000 and 400 cm^−1^. Spectral resolution was set at 4 cm^−1^, sampling 256 scans per sample to obtain high quality spectra (signal to noise ratio values > 4,000 within the 2,100–1,900 cm^−1^ interval). The software OPUS version 6.5 (BRUKER Optics GmbH, Ettlingen, Germany) was used to assess the quality test, subtract the interference of atmospheric CO_2_ and water vapor, correct baseline (rubberband method with 64 points), and to apply vector normalization to the whole spectra.

#### Assessment of Cells Viability

The viability assessment was carried out in parallel with the FTIR analysis to compare the metabolomic alteration with the loss of viability. One hundred microliters of each cells suspension prepared for the FTIR analysis were serial diluted to determine the viable cell counting, in triplicate, on YPDA + chloramphenicol (0.5 g L^−1^) plates. The biocidal effect of the tested compounds was highlighted as cell mortality induced at different concentrations. The cell mortality (M) was calculated as M = (1 – *Cv*/*Ct*) × 100, where *Cv* is the number of viable cells in the tested sample and *Ct* the number of viable cells in the control suspension.

### Statistical Analyses

#### PCA Analysis

Data were analyzed by principal component analysis (PCA), a multivariate statistical unsupervised method, frequently used to reduce complex multidimensional data sets to few principal components. PCA analysis can be applied by using either the entire spectrum, with or without a baseline subtraction, or specific integrated areas. The second method is routinely used (e.g., mass spectrometer elaboration procedures) to reduce noise and minimize the number of variables that permit to obtain clear identification of the species. In this work, we adopted the first method, more common in vibrational spectroscopy applications, for the analysis of the whole frequency range between 3,800 and 600 cm^−1^, except the region between 2,800 and 1,800 cm^−1^, after baseline subtraction and normalization. This method allows to take into account both intensity variation of well-defined peaks and changes in the shape of complex (convoluted) band structures. “prcomp” and “pca2d” open source R routines (www.cran.org) have been used for the PCA analysis.

#### Significant Wavelengths Analysis Throughout the Spectra

In order to show and select the spectral regions with statistically significant differences, an R script (www.cran.org) was employed to reiteratively carry out the following operations:

a. Pairs of spectra, each with at least three replicas, were compared using the Student *t*-test for each wavelength separately, or with a moving average covering 10 wave numbers.
b. For each wave number, the calculated *p*-value was recorded. This operation produced, for each pair of spectra, a vector of *p*-values, that were subsequently transformed in 1 (for *p* < 0.01) and 0 (for *p* > 0.01). These vectors were collected in two types of matrices:
  - One containing the vectors for all possible pairwise comparisons, i.e., the (*n*^2^ – *n*)/2 comparisons among the n conditions under test.
  - One containing only the (*n* – 1) comparisons of the *n* conditions with the control condition.
c. The matrix data were plotted with wave numbers in the x axis and comparisons in the y axis. These plots reported the presence of a wavelength with statistically significant difference (*p* < 0.01) as a dot. The plots were separated by an offset for better visualization.
d. The average number of significant wave numbers were recorded in a square matrix in order to show the percentage of significant differences for each pairwise comparison.

## Results

### Metabolic Performances of M2n and M2n[pBKD2-*Pccbgl1*]-C1 Strains Under Aerobic and Oxygen Limited Conditions

The yeast strain M2n[pBKD2-*Pccbgl1*]-C1, hereafter referred to as C1, was recently engineered to ferment cellobiose into ethanol through the multiple δ-integration of the β-glucosidase *BGL3* gene of *P. chrysosporium* under the constitutive transcriptional control of *PGK1* (Cagnin et al., 2019). The evidence that both parental M2n and recombinant C1 strains displayed similar ethanol yield and growth rate from glucose suggested us that the multiple δ-integration and expression of the *BGL3* genes did not result in any evident metabolic burden when strains were grown in glucose under oxygen limiting conditions (Cagnin et al., 2019). Moreover, aerobic growth determined in microtiter plates on glycerol (1.8%) and the equivalent amount of glucose (2%) showed similar μ_max_ values for both strains, confirming that the integration of multiple copies of this cellulase does not impose metabolic burdens on yeast metabolism in terms of growth performances.

In this study, a more detailed analysis has been carried out to further investigate the physiological status of the recombinant strain C1 by comparing their metabolic performances when exposed to different carbon courses and incubation settings typical of the bioethanol industry. This evaluation takes into account the expected catabolism of the yeast *S. cerevisiae* in a Consolidated BioProcessing (CBP) bioethanol scenario. In this context, a cellobiose-fermenting yeast is likely to start the cellobiose hydrolysis under aerobic conditions and then ferment the resulting glucose. Since the glucose concentration is expected to exceed the Crabtree threshold (around 0.2% glucose), the catabolism will presumably proceed by fermentation even in presence of oxygen (Gombert et al., 2001). Following these considerations, the growth kinetics were carried out aerobically in glycerol to test the respiration conditions and under oxygen-limited conditions in glucose, typical of a cell biomass under intensive CO_2_ flushing.

**Figure 1:**
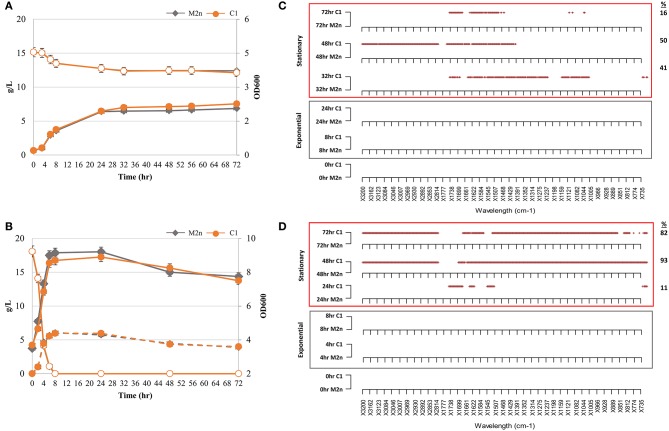
Kinetic and metabolomic patterns of M2n and C1 strains during growth in glycerol and glucose. **(A,B)** Growth kinetics (filled symbols) and glycerol consumption (empty symbols) of M2n and C1 strains **(A)**; growth kinetics (filled symbols), glucose consumption (empty symbols), and ethanol production (dashed lines) of M2n and C1 strains **(B)**. Data shown are the mean values of three replicates and relative standard errors were reported. **(C,D)** FTIR spectroscopy assessment of biochemical variation occurring during exponential (gray box) and stationary (red box) growth in glycerol **(C)** and glucose **(D)**. All significant different wavelengths (*p* < 0.01) between M2n and C1 strains are reported as red dots. The percentages (%) of wavelengths of C1 spectrum significantly different from that of M2n are also reported.

When utilizing glycerol aerobically, both strains showed similar growth kinetics (Figure 1A), suggesting that the δ-integration and expression of the *BGL3* genes did not affect the ability of *S. cerevisiae* C1 to use glycerol as carbon source under aerobic conditions. Once incubated under oxygen-limited settings, glucose consumption of the parental, and recombinant strains did not significantly vary upon the time and growth curves were very similar (Figure 1B). Moreover, both strains produced ethanol levels of nearly 6.00 g/L, corresponding to 66% of the maximum theoretical yield. It was assumed that, the similarity of parameters such as growth rate and yield between strains, was due to the lack of a metabolic burden in the engineered strain.

### Sequencing and Assembly of M2n and C1 Genomes

The likely absence of metabolic burden suggests that no key genes for the catabolism have been deleted or truncated by the δ-integration. To confirm this hypothesis, the genome sequences of both parental and recombinant strains were sequenced combining long single-molecule reads (MiniIon) with short high-quality reads (Illumina) in order to produce robust scaffolds against which the Illumina reads can be mapped to increase the overall assembly quality.

**Figure 2:**
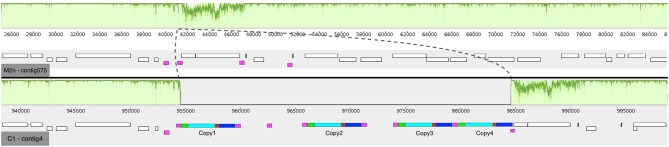
Mauve multi-alignment of the sequences of Chromosome XV of the parental *S. cerevisiae* M2n (contig 575) and the engineered strain C1 (contig 4). The green plots above each sequence show the identity between the two strains. The dashed lines show the inserted region in respect to the M2n parental strain. The white boxes beneath each sequence indicate the annotated genes and the purple ones show the δ-elements of Ty retrotransposon. In the case of the recombinant *S. cerevisiae* C1, the colored boxes show the inserted cassette where green are the promoter sequences, cyan the *BGL3* gene, red the terminator sequences and blue the kanamycin resistance gene.

The average number of paired-end reads (2 × 150 bp) for both strains was 2,605,232, resulting in a 64- and 62-fold genome coverage for *S. cerevisiae* M2n and C1, respectively. In the case of *S. cerevisiae* C1, the number of MinION sequences were 198,892 with an average length of 8,948 bp. The parental genome sequencing gave similar results with 176,882 MinION sequences having an average length of 7,972 bp. For the recombinant *S. cerevisiae* C1, *de novo* assembly generated 23 contigs having a total length of 12.2 Mb, with a N_50_ of 187,462. Notably, 14 chromosomes were assembled in a single contig. Similarly, assembly of the parental *S. cerevisiae* M2n resulted in 28 contigs, having a total length of 12.1 Mb and a N_50_ of 170,717, with 12 chromosomes assembled in a single contig. As reported in Figure 2, four copies of *BGL3* sequence were found in Chromosome XV of the recombinant yeast (see also Table S1). A manual inspection of the recombinant site showed that no alterations occurred in the flanking regions (Figure 2). Additionally, the comparison between the parental and the recombinant genomes did not reveal other major translocations or deletions.

### Metabolomic Fingerprint of Growth Under Aerobic and Oxygen-Limited Conditions in Glycerol and Glucose

FTIR spectra collected along cultivations of both strains in glycerol and glucose were pre-processed and analyzed in order to test the hypothesis that the metabolic burden may be coupled or uncoupled with the metabolomic perturbation. A novel “R” script named SWA (Significant Wavelengths Analysis) has been developed to examine all the statistically relevant differences between pairs of spectra from different experimental conditions. SWA script compares pairs of spectra, each with three or more replicas, using the Student *t*-test for each wavelength separately and produces a plot where all the statistically significant difference wavelengths (*p* < 0.01) are reported as dots. SWA analysis was carried out in order to highlight in which spectral region and, more in detail, at which spectral wavelengths, the metabolomic FTIR fingerprint of recombinant strain was statistically different from that of parental. SWA was performed on IR spectra from both exponential and stationary growth phases to obtain a metabolomic fingerprint of primary and secondary metabolism (Figures 1C,D). Significant differences among spectra were found during the stationary growth phase of the recombinant strain in both glycerol (Figure 1C) and glucose (Figure 1D). Under aerobic growth in glycerol, 41, 50, and 16% wavelengths of C1 spectrum were significantly different from that of M2n at 32, 48, and 72 h, respectively (Figure 1C). The multiple integration and expression of the β-glucosidase *BGL3* genes induced a significant metabolomic alteration during the stationary growth of *S. cerevisiae* C1 in glucose. Metabolomic patterns of Figure 1D displayed an increase percentage of significant different wavelengths between the fingerprints of the two strains shifting from 11% after 24 h to 93 and 82% after 48 and 72 h, respectively. All these differences are detailed in Table S2.

In contrast to growth kinetics data, metabolomic analysis revealed that the expression of the *BGL3* genes produced a significant alteration in cell's physiology during the stationary growth phase. The integration and expression of the *BGL3* genes seems to play a role in the switch between primary and secondary metabolism, changing the quality, and/or the quantity of metabolites produced by the catabolism of the recombinant strain.

Noteworthy, SWA revealed constant patterns of statistically different wavelengths during the stationary growth in glycerol and glucose (Table 2). Specifically, 95 wavelengths in glycerol and 29 wavelengths in glucose were always significantly different between the metabolomic fingerprints of the parental and recombinant strain. These variations affected the Amides (W2) and Mixed (W3) regions and the Amides and Typing (W5) regions in glycerol and glucose, respectively (Table 2). Moreover, wavelengths from 1,638 to 1,611 cm^−1^ (Amide I band components resulting from antiparallel plated sheets and β-turns) and from 1,518 to 1,507 cm^−1^ (Amide II) were stably different between strains regardless of the carbon source or the type of metabolism. These bands, and particularly the Amide I band, has been extensively studied for the role in the secondary structure of proteins (Barth, 2007), suggesting that the integration and/or expression of the *BGL3* genes were somehow linked to protein denaturation.

**Table 2:** Constantly patterns of significant different wavelengths (*p* < 0.01) throughout stationary growth of *S. cerevisiae* C1 in glucose and glycerol under limited-oxygen and aerobic conditions, respectively.

**Incubation**	**Carbon source**	**Spectral Region**	**Wavelengths (cm**^****−1****^**)**		Functional groups^*^
			**From**	**To**	
Aerobiosis	Glycerol	Amides (W2)	1,742	1,711	C = O (1,741);
			1,705	1,699	Amide I
			1,692	1,686	β-turn (1,686)
			1,638		Amide I of β-Sheet
			1,624	1,568	Amide I of β-Sheet
			1,559	1,555	Urea/triglycerides
			1,541	1,501	Amide II (1,540);
		Mixed region (W3)	1,500	1,483	O = C–O^−1^ stretch (1,490);
			1,474	1,470	
			1,458	1,454	CH2 (1,457);
Limited-oxygen conditions	Glucose	Amides (W2)	1,638	1,634	Amide I of β-sheet
			1,626	1,611	Amide I of β-sheet
			1,672		Turns
			1,518	1,507	Shoulder
		Typing region (W5)	723		Phosphate group—Nucleic acid
			716	702	Phosphate group—Nucleic acid

* *Sene et al., 1994; Lasch et al., 2002; Mordehai et al., 2003; Fabian and Naumann, 2004; Yu and Irudayaraj, 2005; Downes et al., 2010; Bellisola and Sorio, 2012; Abidi et al., 2014*.

Differences detected by FTIR analysis in the molecular fingerprint of the two strains during stationary growth could be due to a depletion in cellular energy status in both glycerol and glucose cultivation. Glycerol consumption stopped after 24 h, although more than 12 g/L of this sugar was still available (Figure 1A), and the major metabolomic differences were registered after 32, 48, and 72 h of incubation (Figure 1C). Furthermore, in the case of glucose growth kinetics, significant metabolomic alterations among the strains were detected in cells after 24 h (Figure 1D) when glucose had been already depleted by the strains within 8 h (Figure 1B).

In both glucose and glycerol stationary phases, the cells had a limited, if any, catabolic activity: in the latter case, this is due to the lack of carbon source, whereas in the former case, oxygen limitation is likely the factor constraining energy production. The question remains on why the effect of the residual and limiting energy (or energy rich compounds) was observed in the recombinant strain earlier than in the wild type.

### Metabolic and Metabolomic Evaluation of Recombinant C1 Strain in Cellobiose

**Figure 3:**
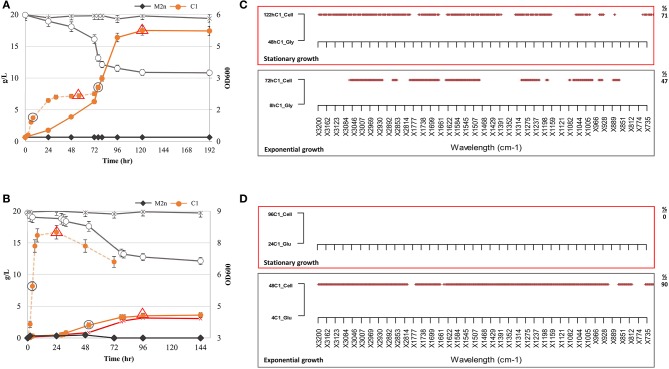
Growth kinetics, ethanol production and metabolic variation of *S. cerevisiae* M2n[pBKD2-*Pccbgl1*]-C1 in cellobiose (2.05%) under aerobic and oxygen-limited conditions. **(A,B)** Growth kinetics (filled symbols). cellobiose consumption (g/L) (empty symbols) and ethanol production (red symbol) of C1 strain under aerobic **(A)** and oxygen limited conditions **(B)**. C1 growth in glycerol and glucose, under aerobic and oxygen limiting conditions, is reported as dashed orange line in **(A,B)**, respectively. The parental M2n yeast, not able to grow in cellobiose, is included as benchmark (dark gray lines). Data shown are the mean values of three replicates and relative standard errors were reported. Gray circle and red triangle symbols indicate middle-exponential and late-stationary phase samples employed for FTIR analysis. **(C,D)** FTIR spectroscopy assessment of biochemical variation occurring during exponential (gray box) and stationary (red box) growth in cellobiose (2.05%) under aerobic and oxygen-limited conditions. SWA analysis was carried out by comparing spectra of the C1 strain in cellobiose with those from the same physiological conditions in **(C)** glycerol and **(D)** glucose. All significant different wavelengths (*p* < 0.01) are reported as red dots. The percentages (%) of spectrum wavelengths of C1 cells grown in cellobiose significantly different from those of C1 cells grown in glycerol or glucose are also reported.

To compare the metabolic performances and the metabolomic profiles during growth on this xenobiotic carbon and energy source, the recombinant C1 strain was incubated in cellobiose under aerobic and oxygen-limiting conditions (Figures 3A,B). As expected, the parental yeast *S. cerevisiae* M2n, included as benchmark, did not grow using this carbon source. On contrary, the engineered C1 strain was able to consume the dimer and grow, confirming the phenotype recently described by our group (Cagnin et al., 2019). Under oxygen-limited conditions, ethanol levels of about 3 g/L were produced, with an ethanol yield corresponding to 79% of the theoretical (0.51 g ethanol per g of consumed glucose equivalent).

The growth of *S. cerevisiae* C1 is not directly comparable with that of wild type on any carbon source, since the concentration of the glucose released from cellobiose remained likely below the Crabtree effect threshold and thus induced a respiration behavior similar to that recorded on glycerol. On the other hand, glycerol utilization, requires a two-step degradation process (Sprague and Cronan, 1977) before entering into glycolysis.

For this reason, SWA analysis compared spectra of the recombinant strain in cellobiose in aerobic and oxygen-limited incubations with those from the same physiological conditions in glycerol and glucose. The analysis has been focused on the middle-exponential and late-stationary growth phases (Figures 3C,D). Once incubated under aerobic conditions, the metabolome of recombinant strain in cellobiose was different from that in glycerol, both at exponential and stationary phases. Differences were observed in all the spectral regions (Table S3) and increased from 47 to 70% of wavelengths from exponential to stationary growth (Figure 3C). Conversely, under oxygen-limited conditions, the footprint of recombinant strain during exponential growth in cellobiose was drastically different from that in glucose (90% significant differences) whereas no variation has been highlighted matching the two metabolomic fingerprints of the stationary phase.

As expected, the detected metabolomic differences are ascribed to the different type and availability of carbon sources. When cells were grown in the presence of oxygen, the availability of glucose is strictly linked to the cellobiose hydrolysis rate and to the time required for glycerol conversion into glucose (Sprague and Cronan, 1977). On contrary, under oxygen-limited conditions (Figure 3D), the two profiles are totally divergent (exponential growth) until stationary phase when glucose has been depleted (Figure 1B) and cellobiose hydrolysis stopped (Figure 3B).

### Stress Response

A FTIR-based assay, already employed for ecotoxicological assessment (Corte et al., 2010; Favaro et al., 2016; Roscini et al., 2019), was carried out to evaluate the type and extent of perturbations induced by stress conditions. Attention was focused on growth conditions typical of lignocellulosic ethanol industry such as high ethanol and lignocellulosic inhibitors concentrations.

#### Effect of Ethanol Exposure on the Intracellular Metabolite Profiles of M2n and C1 Strains

**Figure 4:**
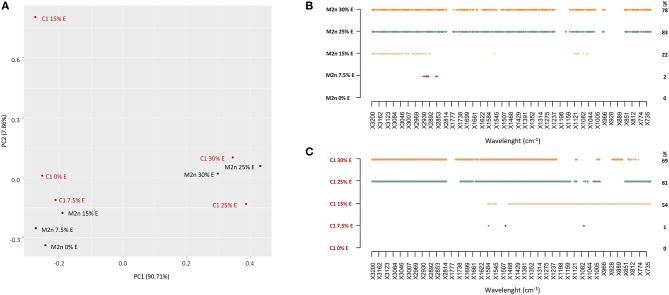
Stress response of *S. cerevisiae* M2n and C1 strains to increasing ethanol concentrations. **(A)** PCA score plot obtained from the IR spectra of M2n (black) and C1 (red) strains at 0, 7.5, 15, 25, and 30% (v/v) ethanol. **(B,C)** SWA patterns of **(B)** M2n and **(C)** C1 strains at 0, 7.5, 15, 25, and 30% (v/v) ethanol. All significant different wavelengths (*p* < 0.01) between M2n and C1 spectra at the tested concentrations and those of the respective controls (cells directly resuspended in water) are reported as dots.

PCA analysis of M2n and C1 metabolomic profiles showed that spectral data variance was mainly distributed according to the first principal component PC1 (90.71%), which clearly separates the spectra at high ethanol concentrations (25 and 30%) from all the other levels (Figure 4A). These data confirmed the outcomes of cell viability analysis indicating that both strains were able to tolerate 7.5 and 15% of alcohol without any viability loss, whereas 100% mortality was observed at 25 and 30% ethanol.

SWA patterns pointed out that the lower ethanol concentration (7.5%) did not significantly alter the metabolome of parental and recombinant strains (Figure 4B). Conversely, 15% ethanol induced different alterations of the metabolomic profiles, evidencing an involvement of the fatty acids region (W1) in the M2n strain, and a widespread effect on all the other regions in the recombinant C1 (Figure 4C; Table S4). These results are in agreement with previous studies describing *S. cerevisiae* response to ethanol. Ethanol exposure has been reported to mainly induce changes in metabolites involved in the metabolism of carbohydrates, lipids and aminoacids. *S. cerevisiae* ethanol-treated cells change the level of fatty acids to decrease membrane fluidity, maintaining the integrity of the plasma membrane (Aguilera et al., 2006). Under ethanol stress, glycolysis was inhibited and changes in the levels of fatty acids and amino acids might confer ethanol tolerance to *S. cerevisiae* (Li et al., 2012). The global perturbation observed at higher ethanol concentrations (25 and 30%) was associated to an increased membrane permeability or to the chemical reactions occurring during cell death (*post-mortem* reaction) (Corte et al., 2015b).

#### Stress Induced by Exposure to Inhibitory Mixtures and Ethanol

FTIR spectroscopy was also used to investigate the response of the parental and recombinant strains to the stress induced by the exposure to increasing concentrations of inhibitory mixtures, with or without 7.5% ethanol.

The four inhibitors mixtures caused similar mortality in the two strains, both with or without 7.5% ethanol: the lower inhibitors concentrations the lower biocidal activity, whereas inhibitors mixtures C and D completely hindered cell viability of both strains (Table 3).

**Table 3:** Mortality (%) induced by four concentrations of inhibitors mixture with or without 7.5% ethanol.

**Strain**	**Ethanol % (v/v)**	**Inhibitor mixtures**			
		**A**	**B**	**C**	**D**
M2n	0	11	39	100	100
C1		14	47	100	100
M2n	7.5	18	74	100	100
C1		20	83	100	100

**Figure 5:**
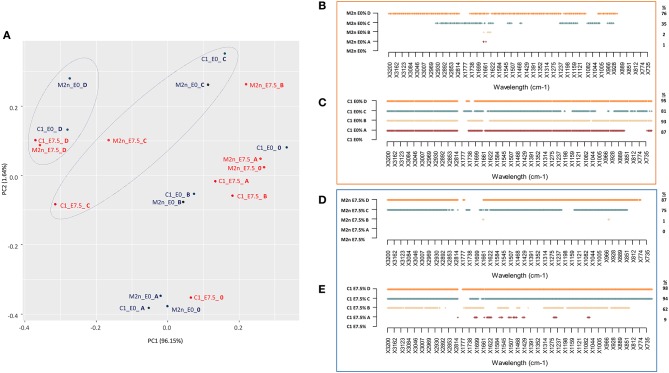
Stress response of *S. cerevisiae* M2n and C1 strains to increasing concentrations of lignocellulosic inhibitors mixtures formulated with 0 or 7.5% of ethanol. **(A)** PCA score plot obtained from the IR spectra of M2n and C1 strains at 0% (blue) and 7.5% (red) ethanol. **(B,C)** SWA patterns of significant wavelengths (*p* < 0.01) throughout spectra of **(B)** M2n and **(C)** C1 cells exposed to increasing concentrations of inhibitor mixture at 0 or 7.5% ethanol, respectively. **(D,E)** SWA patterns of significant wavelengths (*p* < 0.01) throughout spectra of **(D)** M2n and **(E)** C1 cells exposed to increasing concentrations of inhibitor mixture with 0 or 7.5% of ethanol, respectively. The percentages (%) of wavelengths of C1 spectrum significantly different from that of M2n are also reported.

PCA score plot explained 97.69% of the total variance (Figure 5A), mainly accounted by the first principal component PC1 (96.15%). PC1 produced a clear separation of spectra of dead cells (treated with C and D mixtures) from those of living cells (treated with A and B mixtures), with or without ethanol.

SWA patterns allowed to make a more detailed analysis of the response of M2n and C1 strains to these stress conditions (Figures 5B–E). No significant differences were detected between control and M2n stressed cells exposed to mixtures A and B (Figure 5B) whereas, at higher inhibitors concentrations (C and D mixtures), significant spectral alterations were observed (Table S5). On the other hand, the recombinant C1 strain responded differently to these stress conditions, showing a metabolomic reaction that involved all spectral regions and wavelengths already at the lowest inhibitory mixtures concentrations (Table S5), independently from cell viability (Figure 5C). Spectral alterations induced by mixtures C and D corresponds to a post-mortem metabolomic damage (100% mortality), while those induced by A and B represent the active response of yeast cells.

A similar picture has been detected when cells were exposed to 7.5% ethanol and inhibitors mixtures (Figures 5D,E; Table S6). At lower inhibitors concentrations (mixtures A and B), M2n cells did not alter their metabolic activity although showed an increased sensitivity to these stress compounds (Table 3). Conversely, supplementing 7.5% ethanol to the higher inhibitors concentrations (mixtures C and D) resulted in a complete biocidal effect (Table 3) accompanied by an amplified metabolomic reaction (Figure 5D). In the case of *S. cerevisiae* C1, the exposure to ethanol resulted in a different cell reaction, showing an increased mortality, reaching 100% already at mixture B concentration, and a significant decreased metabolomic response to A and B mixtures compared to that observed in absence of ethanol (Figure 5E).

In general, the two strains displayed a different behavior in the presence of these stressful conditions. *S. cerevisiae* M2n seems to react as a resistant strain, coupling low mortality and low metabolomic response (Table 3; Figures 5B–D). On contrary, despite the mortality values similar to those of the parental yeast, the recombinant C1 appears to behave as a sensitive strain, which reacts to stressors changing the quality and/or the quantity of the endo-metabolites produced (Figures 5C–E).

## Discussion

Metabolic burden is a longstanding problem in the engineering of microbes which often leads to undesirable physiological changes (Ding et al., 2018; Wei et al., 2018). In the case of lignocellulosic yeast strains development, physiological responses to metabolic burden are usually evaluated as metabolic performances of the engineered strains such as growth rate, biomass yield and specific substrate consumption rate (Van Rensburg et al., 2012; Ding et al., 2018).

Overall, this study indicates that the multiple δ-integration of a recombinant β-glucosidase gene in Chromosome XV (Figure 2) did not differentially affect the ability of the engineered strain to grow in the presence of different carbon sources both aerobically and under oxygen-limiting conditions (Figures 1A,B, 3A,B). Furthermore, once exposed to increasing concentrations of ethanol and inhibitory compounds, the recombinant strain was found to be as tolerant as the parental yeast (Table 3).

On the contrary, the metabolomic profiles of the recombinant strain were completely altered both under growth (Figures 1, 3C,D) and stress conditions (Figures 4, 5). Under growth conditions, the metabolome of *S. cerevisiae* C1 rapidly changed once the cells entered the stationary phase (Figures 1C,D, 3C,D). This finding could be explained by considering that the production rate of the recombinant enzyme is proportional to the growth rate, implying that proteins and metabolites concentration remained fairly constant. Assuming that the heterologous protein production continued during the stationary phase, an accumulation of the protein can be expected. Similarly, if some metabolites are produced due to the presence of BGL3, it would accumulate for the same reasons described above, likewise to the reported accumulation of an internal inducer due to a *gal7* mutation in *Kluyveromyces lactis* (Cardinali et al., 1997). The hydrolysing activity of BGL3 was indeed mostly found to be evident at stationary phase (Cagnin et al., 2019). As such, during the exponential phase, the synthesis of the recombinant protein achieved a threshold thus accumulating metabolites and shaping differentially the metabolome of the stationary phase. Whether the metabolomic alteration was directly due to BGL3 production or indirectly determined via some yet to discover metabolites, is matter of further investigation already planned in our laboratories.

One possibility to explain this sudden and large metabolomic change is to hypothesize that it is partly due to the alteration induced directly or indirectly by multiple integration and expression of *BGL3* and partly to other alterations to compensate the former ones. In this way, our hypothesis is that the metabolomic change can be dissected in “alterative” and “compensative”, with a null output in terms of metabolic performances. In this sort of altered metabolomic homeostasis, changes induced by the genetic engineering are continuously balanced by compensative metabolomic alterations as in a cantilever, up to reach a final steady state by attenuating the oscillations after each single perturbation. The fact that the metabolomic alterations last throughout the whole stationary phase could indicate that the perturbation is generated continuously, therefore producing an equally continuous compensation. The reasons for choosing the metabolomic fingerprint rather than a full metabolomic analysis is threefold. Firstly, the metabolomic fingerprint gives a holistic view of the metabolome and is well-established as a method to qualify and quantify the stress response of microorganisms (Aguilera et al., 2006; Corte et al., 2010; Mihoubi et al., 2017; Nguyen et al., 2017; Canal et al., 2019). Secondly, fingerprinting approach is suitable for large and complex experimental designs to explore several conditions, the most significant of which could be deeply analyzed by means of metabolomic, transcriptomic, and/or proteomic insights. Finally, the metabolomic fingerprint has less details than the full metabolomics, implying that only relatively gross changes are displayed and that the full “omics” approach will show also changes not detectable at the fingerprint level. This means that, if metabolomic changes are detected with FTIR, they are supposedly rather stable and significant. Further studies dealing with full omics are in progress also to give insights on the differences to be expected by the metabolomic fingerprinting and full “omics” analyses.

## Conclusions

This research indicates that, even in the absence of a metabolic burden, the introduction of a heterologous gene induced huge metabolomic alterations. Considering that four copies of the *BGL3* gene have been integrated into the Chromosome XV without truncating or deleting any parental genes, or promoter sequences, future studies are needed to unveil the mechanisms implied in the hypothesis of alterative and compensative metabolomic changes. Transcriptomic, metabolomics, and proteomic insights will be useful to investigate this fascinating phenomenon and will be instrumental to elucidate the mechanism induced by this δ-integration in an industrial host strain. Beyond the general and speculative interest, this topic is also important at applicative level because a deeper understanding of the interplay between metabolic performances and metabolomic responses is a key factor toward the optimization of protein production in engineered strains for efficient second-generation bioethanol applications.

## Data Availability Statement

The datasets generated for this study can be found at SRA with accession number PRJNA573579 (https://www.ncbi.nlm.nih.gov/bioproject/PRJNA573579).

## Authors Contributions

LCo, LF, and GC contributed to the conception and design of the study. LCa, FD, LR, and LT carried out the experiments. LCo, GC, LF, and SCam performed the statistical analysis. LCo and LF drafted the manuscript. MB, SCas, and WZ critically reviewed the manuscript. All authors contributed to the manuscript revision, read, and approved the submitted version.

### Conflict of Interest

The authors declare that the research was conducted in the absence of any commercial or financial relationships that could be construed as a potential conflict of interest.
