# Supplementary material for: Metabolomic Alterations Do Not Induce Metabolic Burden in the Industrial Yeast M2n[pBKD2-Pccbgl1]-C1 Engineered by Multiple δ-Integration of a Fungal β-Glucosidase Gene
Source: Front Bioeng Biotechnol. 2019 Nov 28;7:376. doi: 10.3389/fbioe.2019.00376 (PMC6893308; doi:10.3389/fbioe.2019.00376)
Supplement: Supplementary file 1 [file Table_1.docx]

**Table S1.** Coordinates of integrated cassette in Chromosome XV of *S. cerevisiae* C1.

| Copy | Gene | Start | end |
| --- | --- | --- | --- |
| Copy1 | *PGK1_P_* | 954644 | 955216 |
|  | *BGL3* | 955224 | 957748 |
|  | *PGK1_T_* | 957746 | 958026 |
|  | *KanMX* | 958027 | 959464 |
| Copy2 | *PGK1_P_* | 966162 | 966728 |
|  | *BGL3* | 966736 | 969261 |
|  | *PGK1_T_* | 969259 | 969541 |
|  | *KanMX* | 969542 | 970976 |
| Copy3 | *PGK1_P_* | 974516 | 975081 |
|  | *BGL3* | 975089 | 977615 |
|  | *PGK1_T_* | 977613 | 977899 |
|  | *KanMX* | 977900 | 979336 |
| Copy4 | *PGK1_P_* | 979698 | 980264 |
|  | *BGL3* | 980272 | 982801 |
|  | *PGK1_T_* | 982799 | 983081 |
|  | *KanMX* | 983082 | 984522 |
