# Supplementary material for: Metabolomic Alterations Do Not Induce Metabolic Burden in the Industrial Yeast M2n[pBKD2-Pccbgl1]-C1 Engineered by Multiple δ-Integration of a Fungal β-Glucosidase Gene
Source: Front Bioeng Biotechnol. 2019 Nov 28;7:376. doi: 10.3389/fbioe.2019.00376 (PMC6893308; doi:10.3389/fbioe.2019.00376)
Supplement: Supplementary file 2 [file Table_2.docx]

**Table S2. S**ignificant different wavelengths (*p* value<0.01) between the metabolomic fingerprints of M2n and C1 strains in glucose and glycerol under limited-oxygen and aerobic conditions, respectively.

| **Physiological condition** | **Carbon source** | **Growth (h)** | **Spectral Region** | **Wavelengths (cm^-1^)** | | **Functional groups*** |
| --- | --- | --- | --- | --- | --- | --- |
|  |  |  |  | *from* | *to* |  |
| **Aerobiosis** | Glycerol | 32 | Amides (W2) | 1742 | 1711 | C=O (1741); |
|  |  |  |  | 1705 | 1699 |  |
|  |  |  |  | 1692 | 1686 | β-turn (1686) |
|  |  |  |  | 1647 | 1642 | Amide (1643) |
|  |  |  |  | 1638 | 1568 | Amide I of β-Sheet |
|  |  |  |  | 1559 | 1555 | urea/triglycerides |
|  |  |  |  | 1545 |  | Amide II |
|  |  |  |  | 1541 | 1500 | Shoulder |
|  |  |  |  |  |  |  |
|  |  |  | Mixed Region (W3) | 1500 | 1225 | O=C-O^-1^ stretch (1490); CH_2_ (1457); C-O-H in-plane bending (1415); C(CH_3_)_2_ / C=O symmetric stretch (1402); (CH_2_)n, C=O (1395); Amide III (1312); P=O asymmetric (1240); PO^2-^ (1125) |
|  |  |  |  |  |  |  |
|  |  |  | Carbohydrates (W4) | 1152 | 1007 | C-O (1140); C-C Skeletal trans conformation (1120); O-P-O (1095); P=O asymmetric (1085); C-C Skeletal cis conformation (1076); C-O-P (1050); C-O-H (1028) |
|  |  |  |  |  |  |  |
|  |  |  | Typing Region (W5) | 727 | 720 |  |
|  |  |  |  | 710 | 702 |  |
|  |  |  |  |  |  |  |
|  |  | 48 | Fatty Acids (W1) | 3200 | 2800 | CH_3_(2957, 2872), CH_2_(2920, 2852), C-H (3077), N-H (3084) |
|  |  |  |  |  |  |  |
|  |  |  | Amides (W2) | 1757 | 1672 | C=O (1741); C=O H-bonded (1708); β-turn (1685); β-sheet (1684) |
|  |  |  |  | 1667 | 1636 | α-helix (1658); Amide I of α-helical structure (1655); Amide I (1643); Amide I of β-Sheet (1638) |
|  |  |  |  | 1624 | 1501 | Amide I of β-Sheet (1624); Amide II (1540); shoulder |
|  |  |  |  |  |  |  |
|  |  |  | Mixed Region (W3) | 1499 | 1410 | O=C-O^-^ stretch (1490); CH_2_ (1457); C-O-H in-plane bending (1415) |
|  |  |  |  | 1406 | 1402 | C(CH_3_)_2_ / C=O symmetric stretch (1402); |
|  |  |  |  | 1399 | 1395 | (CH_2_)n, C=O (1395); |
|  |  |  |  |  |  |  |
|  |  | 72 | Amides (W2) | 1774 | 1674 | C=O (1741); C=O H-bonded (1708); β-turn (1685); β-sheet (1684) |
|  |  |  |  | 1638 |  | Amide I of β-Sheet (1638) |
|  |  |  |  | 1632 | 1566 | Amide I of β-Sheet (1624); Amide II (1540); |
|  |  |  |  | 1559 | 1547 | Amide II |
|  |  |  |  | 1541 | 1501 | Shoulder |
|  |  |  |  |  |  |  |
|  |  |  | Mixed Region (W3) | 1499 | 1483 | O=C-O^-^ stretch (1490) |
|  |  |  |  | 1474 | 1470 |  |
|  |  |  |  | 1458 | 1454 | CH_2_ |
|  |  |  |  |  |  |  |
|  |  |  | Carbohydrates (W4) | 1115 | 1113 |  |
|  |  |  |  | 1098 | 1096 | O-P-O |
|  |  |  |  | 1038 | 1032 |  |
|  |  |  |  |  |  |  |
| **Under oxygen- limited condition** | Glucose | 24 | Amides (W2) | 1746 | 1672 | C=O (1741); Amide I band components resulting from antiparallel plated sheets and β-turns (1695); |
|  |  |  |  | 1638 | 1611 | Amide I of β-Sheet |
|  |  |  |  | 1545 | 1507 | Amide II |
|  |  |  |  |  |  |  |
|  |  |  | Typing Region (W5) | 725 | 723 |  |
|  |  |  |  | 718 | 702 |  |
|  |  |  |  |  |  |  |
|  |  | 48 | Fatty Acids (W1) | 3200 | 2801 | CH_3_(2957, 2872), CH_2_(2920, 2852), C-H (3077), N-H (3084) |
|  |  |  |  |  |  |  |
|  |  |  | Amides (W2) | 1696 | 1501 | Amide I band components resulting from antiparallel plated sheets and β-turns (1695); β-turn (1685); β-sheet (1684); Amide I of β-Sheet (1638); Amide II (1540); |
|  |  |  |  |  |  |  |
|  |  |  | Mixed Region (W3) | 1499 | 1200 | O=C-O- stretch (1490); CH_2_ (1457); C-O-H in-plane bending (1415); C(CH_3_)_2_ / C=O symmetric stretch (1402); (CH_2_)n (1395) |
|  |  |  |  |  |  |  |
|  |  |  | Carbohydrates (W4) | 1198 | 901 | C-O ring (1180); C-O-P, C-C C-O-H (1160); P=O asymmetric stretch (1085); C-C Skeletal cis conformation (1076); C-O-P (1050); Glycogen (1029); C-O-H (1028); ribose, uracil (994) |
|  |  |  |  |  |  |  |
|  |  |  | Typing Region (W5) | 899 | 702 | deoxyribose ring (899); CO32-(874); Cytosine ring vibration (788); DNA peak (785); |
|  |  |  |  |  |  |  |
|  |  | 72 | Fatty Acids (W1) | 3200 | 2801 | CH_3_(2957, 2872); CH_2_(2920, 2852); C-H (2077); N-H (2084) |
|  |  |  |  |  |  |  |
|  |  |  | Amides (W2) | 1672 | 1634 | C=N (1670), Amide I of β-Sheet (1638) |
|  |  |  |  | 1626 | 1543 | Amide I of β-Sheet (1624); Amide II (1543) |
|  |  |  |  | 1518 | 1501 |  |
|  |  |  |  |  |  |  |
|  |  |  | Mixed Region (W3) | 1499 | 1200 | O=C-O- stretch (1489); C-O-H in-plane bending (1415); C(CH_3_)_2_ / C=O symmetric stretch (1402); (CH_2_) _n_ (1395); |
|  |  |  |  |  |  |  |
|  |  |  | Carbohydrates (W4) | 1198 | 901 | C-O-P; C-C; C-O-H (1160); C-C Skeletal trans conformation (1120); C-O (1100); O-P-O (1095); P=O asymmetric (1085); C-C Skeletal cis conformation (1076); C-O-P (1050); C-O-H (1028); ribose, uracil (994); (PO_3_)^2-^ (965); C-C (962); P-O (950); C-O-P (920); ribose ring (916) |
|  |  |  |  |  |  |  |
|  |  |  | Typing Region (W5) | 899 | 858 | deoxyribose ring (899); CO_3_^2-^ (874) |
|  |  |  |  | 826 | 793 | O-P-O (813); RNA peak (811) |
|  |  |  |  | 774 | 772 |  |
|  |  |  |  | 743 |  |  |
|  |  |  |  | 723 | 722 |  |
|  |  |  |  | 716 | 702 |  |
|  |  |  |  |  |  |  |

*(Sene et al., 1994;Lasch et al., 2002;Mordehai et al., 2003;Fabian and Naumann, 2004;Yu and Irudayaraj, 2005;Downes et al., 2010;Bellisola and Sorio, 2012;Corte et al., 2012;Abidi et al., 2014).
