# Supplementary material for: Metabolomic Alterations Do Not Induce Metabolic Burden in the Industrial Yeast M2n[pBKD2-Pccbgl1]-C1 Engineered by Multiple δ-Integration of a Fungal β-Glucosidase Gene
Source: Front Bioeng Biotechnol. 2019 Nov 28;7:376. doi: 10.3389/fbioe.2019.00376 (PMC6893308; doi:10.3389/fbioe.2019.00376)
Supplement: Supplementary file 3 [file Table_3.docx]

**Table S3. S**ignificant wavelengths between the metabolomic fingerprints of the recombinant strain in in aerobic (cellobiose vs glycerol) and oxygen-limited incubations (cellobiose vs glucose).

| **Physiological condition** | **Carbon source** | **Growth phase** | **Spectral Region** | **Wavelengths** | | **Functional** |
| --- | --- | --- | --- | --- | --- | --- |
|  |  |  |  | **(cm^-1^)** | | **groups*** |
|  |  |  |  | *from* | *to* |  |
| **Aerobiosis** | **Cellobiose** | **Exponential** | Fatty Acids (W1) | 3063 | 3061 |  |
|  | vs | **8h vs 72h** |  | 3057 | 2913 |  |
|  | **Glycerol** |  |  | 2868 | 2847 | CH2 (2852) |
|  |  |  |  |  |  |  |
|  |  |  | Amides (W2) | 1794 | 1663 | Amide I -β-turn (1686)- turns (1672) |
|  |  |  |  | 1634 | 1500 | Amide I of β-Sheet (1638) -urea/triglycerides (1559-1555)- Amide II (1540) |
|  |  |  |  |  |  |  |
|  |  |  | Mixed Region (W3) | 1500 | 1481 | O=C-O^-1^ stretch |
|  |  |  |  | 1296 | 1215 |  |
|  |  |  |  |  |  |  |
|  |  |  | Carbohydrates (W4) | 1186 | 1175 |  |
|  |  |  |  | 1082 | 1076 | P=O asymmetric (1085); C-C Skeletal cis conformation (1076); |
|  |  |  |  | 1065 | 976 | C-O-P (1050); C-O-H (1028) |
|  |  |  |  | 951 | 934 |  |
|  |  |  |  |  |  |  |
|  |  |  | Typing Region (W5) | 887 | 858 |  |
|  |  |  |  |  |  |  |
|  |  | **Stationary** | Fatty Acids (W1) | 3200 | 2803 | CH3(2957, 2872), CH2(2920, 2852), C-H (3077), N-H (3084) |
|  |  | **48h vs 122h** |  |  |  |  |
|  |  |  | Amides (W2) | 1800 | 1790 |  |
|  |  |  |  | 1752 | 1663 | C=O (1741); Amide I -β-turn (1686); turns (1672) |
|  |  |  |  | 1628 | 1500 | urea/triglycerides (1559-1555); Amide II (1540) |
|  |  |  |  |  |  |  |
|  |  |  | Mixed Region (W3) | 1500 | 1385 | O=C-O^-1^ stretch (1490); CH2 (1457); C-O-H in-plane bending (1415); C(CH3)_2_ / C=O symmetric stretch (1402); (CH2)n, C=O (1395); |
|  |  |  |  | 1323 | 1200 | Amide III (1312); P=O asymmetric (1240); |
|  |  |  |  |  |  |  |
|  |  |  | Carbohydrates (W4) | 1200 | 1161 |  |
|  |  |  |  | 1053 | 990 | C-O (1140); C-C Skeletal trans conformation (1120); O-P-O (1095); P=O asymmetric (1085); C-C Skeletal cis conformation (1076); C-O-P (1050); C-O-H (1028) |
|  |  |  |  | 949 | 928 |  |
|  |  |  |  |  |  |  |
|  |  |  | Typing Region (W5) | 882 | 878 |  |
|  |  |  |  | 754 | 702 |  |
|  |  |  |  |  |  |  |
| **Under limited-oxygen** | **Cellobiose** | **Exponential** | Fatty Acids (W1) | 3200 | 2800 | CH3(2957, 2872), CH2(2920, 2852), C-H (3077), N-H (3084) |
| **condition** | vs | **4h vs 48h** |  |  |  |  |
|  | **Glucose** |  | Amides (W2) | 1767 | 1657 | C=O (1741); C=O H-bonded (1708); β-turn (1685); β-sheet (1684) |
|  |  |  |  | 1642 | 1638 | α-helix (1658); Amide I of α-helical structure (1655); Amide I (1643); Amide I of β-Sheet (1638) |
|  |  |  |  | 1634 | 1500 | Amide I of β-Sheet (1624); Amide II (1540); shoulder |
|  |  |  |  |  |  |  |
|  |  |  | Mixed Region (W3) | 1500 | 1200 | O=C-O- stretch (1490); CH2 (1457); C-O-H in-plane bending (1415); C(CH3)2 / C=O symmetric stretch (1402); (CH2)n, C=O (1395); |
|  |  |  |  |  |  |  |
|  |  |  | Carbohydrates (W4) | 1200 | 907 |  |
|  |  |  |  |  |  |  |
|  |  |  | Typing Region (W5) | 862 | 801 |  |
|  |  |  |  | 743 | 702 |  |
|  |  |  |  |  |  |  |

*(Sene et al., 1994;Lasch et al., 2002;Mordehai et al., 2003;Fabian and Naumann, 2004;Yu and Irudayaraj, 2005;Downes et al., 2010;Bellisola and Sorio, 2012;Corte et al., 2012;Abidi et al., 2014).
