# Supplementary material for: Metabolomic Alterations Do Not Induce Metabolic Burden in the Industrial Yeast M2n[pBKD2-Pccbgl1]-C1 Engineered by Multiple δ-Integration of a Fungal β-Glucosidase Gene
Source: Front Bioeng Biotechnol. 2019 Nov 28;7:376. doi: 10.3389/fbioe.2019.00376 (PMC6893308; doi:10.3389/fbioe.2019.00376)
Supplement: Supplementary file 4 [file Table_4.docx]

**Table S4.** **S**ignificant different wavelengths (*p* value<0.01) between the metabolomic fingerprints of M2n and C1 strains under ethanol stress.

| **Ethanol (%)** | **Strain** | **Spectral Region** | **Wavelengths** | | **Functional** |
| --- | --- | --- | --- | --- | --- |
|  |  |  | **(cm^-1^)** | | **groups*** |
|  |  |  | *from* | *to* |  |
| **7.5** | M2n | Fatty Acids (W1) | 2930 | 2926 | CH_3_ and CH_2_ asymmetric stretch |
|  |  |  | 2922 | 2917 | CH_2_ asymmetric stretch |
|  |  |  | 2909 | 2899 |  |
|  |  |  | 2859 | 2855 | CH_2_ symmetric stretch |
|  |  |  |  |  |  |
| **15** |  | Fatty Acids (W1) | 3200 | 3032 | CH_3_, CH_2_ and CH stretch |
|  |  |  | 3023 | 3015 | CH_3_ and CH_2_ asymmetric stretch |
|  |  |  | 3009 | 3000 | CH_3_ and CH_2_ asymmetric stretch |
|  |  |  | 2990 | 2907 | CH_3_ and CH_2_ asymmetric stretch |
|  |  |  | 2895 | 2890 | CH_2_ symmetric stretch |
|  |  |  |  |  |  |
|  |  | Amides (W2) | 1549 | 1547 | Amide II |
|  |  |  |  |  |  |
|  |  | Carbohydrates (W4) | 1127 | 1109 | C-C Skeletal *trans* conformation  stretching |
|  |  |  | 1103 | 1098 | Nucleic Acids O-P-O stretching |
|  |  |  | 1076 | 1073 | C-C Skeletal cis conformation stretching |
|  |  |  | 1055 | 1051 | C-O-P stretching |
|  |  |  |  |  |  |
| **7.5** | C1 | Amides (W2) | 1578 | 1576 | Aminoacid side chain absorptions (N-H e C-C) |
|  |  |  |  |  |  |
|  |  | Mixed Region (W3) | 1489 | 1487 | O=C-O^-^ stretch |
|  |  |  |  |  |  |
|  |  | Carbohydrates (W4) | 1071 | 1069 | C-C Skeletal cis conformation stretching |
|  |  |  |  |  |  |
| **15** |  | Amides (W2) | 1584 | 1564 | Aminoacid side chain absorptions (N-H e C-C) |
|  |  |  | 1561 | 1549 | N-H deformation in Amide II |
|  |  |  | 1545 | 1537 | Amide II |
|  |  |  |  |  |  |
|  |  | Mixed Region (W3) | 1472 | 1200 | (N-H), (C-N), (C=0), (C-C) and (CH_3_) stretching in Amide III |
|  |  |  |  |  |  |
|  |  | Carbohydrates (W4) | 1200 | 900 | Carbohydrates |
|  |  |  |  |  |  |
|  |  | Typing Region (W5) | 900 | 735 | C=C, C=N, C—H in nucleotide ring structure |
|  |  |  | 731 | 706 | C=C, C=N, C—H in nucleotide ring structure +  CH_2_ rocking |
|  |  |  |  |  |  |

*(Sene et al., 1994;Lasch et al., 2002;Mordehai et al., 2003;Fabian and Naumann, 2004;Yu and Irudayaraj, 2005;Downes et al., 2010;Bellisola and Sorio, 2012;Corte et al., 2012;Abidi et al., 2014).
