# Supplementary material for: Metabolomic Alterations Do Not Induce Metabolic Burden in the Industrial Yeast M2n[pBKD2-Pccbgl1]-C1 Engineered by Multiple δ-Integration of a Fungal β-Glucosidase Gene
Source: Front Bioeng Biotechnol. 2019 Nov 28;7:376. doi: 10.3389/fbioe.2019.00376 (PMC6893308; doi:10.3389/fbioe.2019.00376)
Supplement: Supplementary file 5 [file Table_5.docx]

**Table S5.** **S**ignificant different wavelengths (*p* value<0.01) between the metabolomic fingerprints of M2n and C1 strains under stress induced by inhibitory mixtures.

| **Physiological condition** | **STRAIN** | **Spectral Region** | **Wavelengths** | | **Functional** |
| --- | --- | --- | --- | --- | --- |
|  |  |  | **(cm^-1^)** | | **groups*** |
|  |  |  | *from* | *to* |  |
| **ETHANOL 0% A** | **M2n** | Fatty Acids (W1) | 2801 |  | C-H stretching |
|  |  |  |  |  |  |
|  |  | Amides (W2) | 1753 | 1752 | C=O stretch |
|  |  |  | 1665 | 1659 | C=N stretch |
|  |  |  | 1649 |  | Amide I α-helical structure |
|  |  |  |  |  |  |
|  |  |  |  |  |  |
| **ETHANOL 0% B** |  | Amides (W2) | 1757 | 1721 | C=O stretch |
|  |  |  | 1661 |  | C=N stretch |
|  |  |  | 1645 | 1624 | Amide I |
|  |  |  |  |  |  |
| **ETHANOL 0% C** |  | Fatty Acids (W1) | 3055 | 3044 | C-H stretching |
|  |  |  | 3027 | 2990 | Stretching C-H aromatic rings |
|  |  |  | 2986 | 2982 | CH_3_ and CH_2_ asymmetric stretch |
|  |  |  | 2955 | 2953 | CH_3_ asymmetric stretch |
|  |  |  | 2946 | 2801 | CH_3_ and CH_2_ symmetric and asymmetric stretch |
|  |  |  |  |  |  |
|  |  | Amides (W2) | 1738 | 1696 | C=O stretch |
|  |  |  | 1644 | 1615 | Amide I of β-sheet |
|  |  |  | 1221 | 1200 | PO_2_^-^ stretching |
|  |  |  |  |  |  |
|  |  | Carbohydrates (W4) | 1200 | 1157 | C=O and C-OH stretching |
|  |  |  | 966 | 928 | (PO_3_)^2-^ stretching |
|  |  |  |  |  |  |
| **ETHANOL 0% A** | **C1** | Fatty Acids (W1) | 3200 | 2801 | (C-H)_n_ stretching |
|  |  |  |  |  |  |
|  |  | Amides (W2) | 1759 | 1661 | CH_2_-COO- ν(C=O) of phospholipid esters |
|  |  |  | 1644 | 1500 | Amide I and Amide II |
|  |  |  |  |  |  |
|  |  | Mixed Region (W3) | 1500 | 1200 | (N-H), (C-N), (C=0), (C-C) and (CH_3_) stretching in Amide III |
|  |  |  |  |  |  |
|  |  | Carbohydrates (W4) | 1200 | 900 | C-O-C and C-O vibration in ring structure |
|  |  |  |  |  |  |
|  |  | Typing Region (W5) | 900 | 723 | C=C, C=N, C—H in nucleotide ring structure |
|  |  |  | 716 | 702 | CH_2_ rocking |
|  |  |  |  |  |  |
| **ETHANOL 0% B** |  | Fatty Acids (W1) | 3200 | 2801 | (C-H)_n_ stretching |
|  |  |  |  |  |  |
|  |  | Amides (W2) | 1796 | 1792 | C=O stretch in Amide I |
|  |  |  | 1725 | 1661 | CH_2_-COO- ν(C=O) of phospholipid esters |
|  |  |  | 1638 | 1500 | Amide I and Amide II |
|  |  |  |  |  |  |
|  |  | Mixed Region (W3) | 1500 | 1200 | (N-H), (C-N), (C=0), (C-C) and (CH_3_) stretching in Amide III |
| **ETHANOL 0% C** |  |  |  |  |  |
|  |  | Carbohydrates (W4) | 1200 | 953 | C-O-C and C-O vibration in ring structure |
|  |  |  | 936 | 900 | DNA backbone stretching |
|  |  |  |  |  |  |
|  |  | Typing Region (W5) | 900 | 833 | C=C, C=N, C—H in nucleotide ring structure |
|  |  |  | 822 | 818 | RNA backbone stretching |
|  |  |  | 812 | 806 | C=C, C=N, C—H in nucleotide ring structure |
|  |  |  |  |  |  |

*(Sene et al., 1994;Lasch et al., 2002;Mordehai et al., 2003;Fabian and Naumann, 2004;Yu and Irudayaraj, 2005;Downes et al., 2010;Bellisola and Sorio, 2012;Corte et al., 2012;Abidi et al., 2014).
