# Supplementary material for: Metabolomic Alterations Do Not Induce Metabolic Burden in the Industrial Yeast M2n[pBKD2-Pccbgl1]-C1 Engineered by Multiple δ-Integration of a Fungal β-Glucosidase Gene
Source: Front Bioeng Biotechnol. 2019 Nov 28;7:376. doi: 10.3389/fbioe.2019.00376 (PMC6893308; doi:10.3389/fbioe.2019.00376)
Supplement: Supplementary file 6 [file Table_6.docx]

**Table S6.** **S**ignificant different wavelengths (*p* value<0.01) between the metabolomic fingerprints of M2n and C1 strains under stress induced by inhibitory mixtures at ethanol concentration of 7.5%.

| **Physiological condition** | **STRAIN** | **Spectral Region** | **Wavelengths** | | **Functional** |
| --- | --- | --- | --- | --- | --- |
|  |  |  | **(cm^-1^)** | | **groups*** |
|  |  |  | *from* | *to* |  |
| **ETHANOL 7.5% B** | **M2n** | Amides (W2) | 1663 | 1655 | C=N stretch |
|  |  |  |  |  |  |
| **ETHANOL 7.5% A** |  | Fatty Acids (W1) | 3200 | 2801 | (C-H)_n_ stretching |
|  |  |  |  |  |  |
|  |  | Amides (W2) | 1800 | 1796 | Nucleic acid bases |
|  |  |  | 1750 | 1672 | CH_2_-COO- ν(C=O) of phospholipid esters |
|  |  |  | 1665 |  | CH_2_-COO- ν(C=O) of phospholipid esters |
|  |  |  | 1661 | 1651 | C=N stretch |
|  |  |  | 1642 | 1603 | Amide I of β-sheet |
|  |  |  | 1576 |  | COO^-^ asymmetric stretch |
|  |  |  | 1568 |  | Aminoacids side chains vibrations |
|  |  |  | 1559 | 1549 | C=N stretching |
|  |  |  | 1539 |  | Amide II |
|  |  |  | 1520 | 1500 | Amide II: N-H deformation + C-N stretching |
|  |  |  |  |  |  |
|  |  | Mixed Region (W3) | 1500 | 1491 | (N-H), (C-N) in Amide III |
|  |  |  |  |  |  |
|  |  | Carbohydrates (W4) | 1277 | 1242 | C-OH stretching in -COOH group |
|  |  |  |  |  |  |
| **ETHANOL 7.5% B** | **C1** | Amides (W2) | 1771 | 1655 | CH_2_-COO- ν(C=O) of phospholipid esters |
|  |  |  | 1572 | 1520 | COO- asymmetric stretch +  Aminoacids side chains vibrations |
|  |  |  |  |  |  |
|  |  | Mixed Region (W3) | 1483 | 1200 | (N-H), (C-N), (C=0), (C-C) and (CH_3_) stretching in Amide III |
|  |  |  |  |  |  |
|  |  | Carbohydrates (W4) | 1200 | 953 | C-O-C and C-O vibration in ring structure |
|  |  |  | 934 | 932 | DNA backbone stretching |
|  |  |  | 926 | 900 | DNA backbone stretching |
|  |  |  |  |  |  |
|  |  | Typing Region (W5) | 900 | 801 | C=C, C=N, C—H in nucleotide ring structure |
|  |  |  |  |  |  |

*(Sene et al., 1994;Lasch et al., 2002;Mordehai et al., 2003;Fabian and Naumann, 2004;Yu and Irudayaraj, 2005;Downes et al., 2010;Bellisola and Sorio, 2012;Corte et al., 2012;Abidi et al., 2014).
